# Accelerometry-based assessment of physical activity and sedentary behavior in adult survivors of childhood acute lymphoblastic leukemia and their healthy peers

**DOI:** 10.1038/s41598-023-34689-5

**Published:** 2023-05-08

**Authors:** Tomáš Vyhlídal, Jan Dygrýn, Jaroslava Hrubá, František Chmelík

**Affiliations:** Faculty of Physical Culture, Palacký University Olomouc, třída Míru 117, 771 11 Olomouc, Czech Republic

**Keywords:** Cancer, Health care, Oncology

## Abstract

Adult survivors of childhood acute lymphoblastic leukemia (ASALL) compose a specific group that faces an increased risk of experiencing late effects of their earlier treatment. Physical activity (PA) may be one of the appropriate means for preventing or minimizing the late effects of treatment. The main purpose of this study is to characterize device-measured PA and sedentary behavior (SB) among ASALL. The specific objective was to compare the movement behavior with a group recruited from the healthy population and to determine the degree of compliance with health recommendations for PA in the adult population. Twenty ASALL and 21 healthy control group (CG) members participated in the study. Participants were between 18 and 30 years old. Movement behavior was assessed for seven days using an Axivity AX3 accelerometer and a 24-h wearing protocol. Movement behavior was characterized by the amount of time spent in SB, light PA (LPA), moderate PA (MPA), and vigorous PA (VPA). There were no significant differences in movement behavior or compliance with PA recommendations between the ASALL and CG. During the week, the ASALL accumulated 711 min per day of SB vs. 636 min per day in the CG (p = 0.26); the ASALL had 186 min per day of LPA vs. 201 min per day in the CG (p = 0.47); the ASALL had 132 min per day of MPA vs. 147 min per day in the CG (p = 0.25); and the ASALL had 5 min per day of VPA vs. 4 min per day in the CG (p = 0.48). All research participants (ASALL and CG) met the PA recommendations of > 150 min per week for moderate PA. The results of our study suggest that ASALL, even after suffering from that disease in childhood, display comparable levels of PA and SB to their healthy peers. Both groups met the health recommendations for PA. The device-based monitoring of PA and SB should be an integral part of monitoring the late effects of treatment.

## Introduction

Acute lymphoblastic leukemia is the most common childhood malignancy in pediatric patients^1^. Depending on the success of treatment in childhood, a specific group of young adults who may be at risk of late effects of treatment is gradually formed^2,3^. The most common late effects of treatment include, for example, premature mortality, secondary malignancy, neurological/neurocognitive disorders, cardiotoxicity, endocrine disorders (growth hormone deficiency, precocious puberty and obesity), bone disorders (osteoporosis and osteonecrosis) and social/psychological disorders^4,5^.

Adult survivors of childhood acute lymphoblastic leukemia (ASALL) are monitored in the Czech Republic at a Dispensary Outpatient Clinic. This outpatient clinic provides highly specialized preventive and curative care to patients and continuously monitors and evaluates the occurrence of possible late effects of cancer and its treatment. At present, however, monitoring is not focused on the area of physical activity (PA), which is one of the appropriate ways of preventing or minimizing the late effects of treatment^6,7^.

Regular PA in surviving patients may improve their quality of life^1^, cardiorespiratory fitness^8^, and neurocognitive functions^3^ and reduce weight gain^9^. In contrast, sedentary behavior (SB) may increase the harmful effects of treatment, the incidence of obesity, and the development of metabolic syndrome or cardiovascular disease in this target group^10,11^. Nevertheless, it can be expected that a decrease in PA and a failure to achieve World Health Organization PA recommendations can occur in ASALL, although PA is very desirable for this target group^7,12^. There is a lack of studies which have evaluated the comprehensive device-measured movement behavior (including SB) of this target group to obtain a better understanding of the topic, even though it is absolutely necessary^1^. Many studies identify and describe PA on the basis of subjective questioning (self-report) of the target group^5–7,13^. It can be assumed that the subjective and device-based assessment of PA can vary greatly. Compared with other types of studies, self-reported data may lead to an overestimation of the level of PA^14^ and, conversely, an underestimation of SB^15^.

For this reason, our study focused on device-based monitoring and evaluation of PA and SB. The main objective of this study was to characterize the device-measured PA and SB of young adults who were diagnosed with acute lymphoblastic leukemia in childhood. The specific objective was to compare their movement behavior with a group of healthy controls and to determine to what extent the target group met the recommendations for PA for the adult population defined by the World Health Organization^16^.

## Methods

### Design and participants

Data from 20 ASALL and 21 of their healthy peers (control group (CG)) were evaluated in this cross-sectional study. Inclusion criteria for the ASALL group were as follows: age between 18 and 30 years; underwent active treatment for acute lymphoblastic leukemia in childhood at the departments of pediatric hematology and hematology-oncology in the Czech Republic. Exclusion criteria included a disability or health condition that was unrelated to the acute lymphoblastic leukemia treatment. In ASALL, treatment was discontinued in childhood. In the case of the CG, those who were aged 18–30 years and residents of the Czech Republic were included. Exclusion criteria included having a disability or a health condition. Health condition was defined as the presence of any acute or chronic medical conditions that would compromise their ability to participate in the study.

### Recruitment of participants

The study focused on ASALL, which is a very specific population of young adults who had acute lymphoblastic leukemia in childhood. In the Czech Republic, these former patients were not officially registered anywhere in the public health systems. One of the main ways in which our study team could reach them was through patient organizations, where they had previously registered based on their own interest. Two collaborating patient organizations (“Společně k úsměvu” and “Sdružení Šance”) provided us with contact details of ASALL who met the inclusion criteria of our study. We approached all these ASALL (n = 31) and offered them the opportunity to participate in the study. Twenty-one of them expressed interest in participating in the study (67.7% response rate), and we were able to obtain valid data from 20 of them to include in the study (accelerometer data from 1 participant were not valid). We also asked each ASALL participant to include one of their peers in the study. The choice of peers depended solely on ASALL. In this way, we obtained valid data from 21 members of the CG. No randomization procedure was applied to recruit participants. Power calculation^17^ indicated that the required sample size per group was 20 (https://homepage.univie.ac.at/robin.ristl/samplesize.php; P(X > Y) = 0.76; Alpha two-sided = 0.05; Power = 0.8).

### Data collection

The data collection took place on June 14–20, 2021, and lasted for seven days. The monitoring devices, as well as all necessary instructions, were handed over to the ASALL and CG members in person. Upon completion of the monitoring, the participants sent the devices back by mail (at the researchers’ expense) to the principal investigator of the project for evaluation. Data were collected over a habitual week (5 working days and 2 weekend days) with no public holidays and no participants on vacation/leave in the ASALL or in the CG. The research took place after the end of all restrictions imposed during the second wave of the COVID-19 pandemic in the Czech Republic. The daily routine of the research participants was therefore not disturbed in any way, and all activities could be carried out without any limitations.

Before the data collection started, the research participants were informed about the entire research study, the data collection process, and the method used for evaluation. Information on marital status, occupational status, age, height, weight, onset of the disease, and length of the treatment was obtained from the participants by a questionnaire survey.

### Physical activity and sedentary behavior

Device-measured movement behavior, including SB, light PA (LPA), moderate PA (MPA), and vigorous PA (VPA), was assessed using Axivity AX3 accelerometers (Axivity Ltd, Newcastle, UK). Axivity AX3 is considered to be equivalent to GENEActiv^18^ and was used for the monitoring of movement behavior in > 100,000 adults in the UK Biobank study^19^. The participants were instructed to wear the accelerometer on their nondominant wrist continuously for up to seven days (24-h wearing protocol) and only remove it for activities involving water. The accelerometers were initialized to collect data on three axes at a frequency of 100 Hz with a dynamic range of ± 8 g, which was downloaded using Open Movement software (Open Lab, Newcastle University, UK). Following Miguels et al.^20^, the open source R package GGIR (v2.1-0, https://cran.r-project.org/web/packages/GGIR/) was used to analyze the .cwa files. To categorize the time spent on movement behaviors, we used the proposed Euclidean Norm Minus One thresholds for SB: < 45 mg^21^, LPA: ≥ 45 and < 100 mg, MPA ≥ 100 mg, and VPA ≥ 428 mg^22^. To calculate the total time spent in each movement behavior (SB, LPA, MPA, and VPA), we used the default setting of summing up 5-s epochs that met the threshold criteria^20^. For inclusion in the analysis, participants were required to wear the accelerometer for at least 4 valid days, including at least 1 weekend day. A day was considered valid if the participant wore the accelerometer for at least 16 h, if there were available wear data for each 15-min interval in the 24-h cycle (after imputation)^20^, and if the accelerometer was worn for at least 8 h during the waking period.

According to the World Health Organization guidelines for PA for adults (aged 18–64), weekly volumes of 150–300 min of moderate intensity, 75–150 min of vigorous intensity, or an equivalent combination of MVPA^16^ were set as the PA criteria. For the purposes of this study, the definition of compliance was set at > 150 min per week for moderate intensity or > 75 min per week for vigorous intensity.

### Statistical data processing

IBM SPSS Statistics 25 statistical software (IBM SPSS, Inc. Chicago, IL, USA) was used. Descriptive statistics (median, interquartile range) were used to provide the basic characteristics of the study sample. To compare the groups (sex, treatment), the nonparametric Mann‒Whitney U test was used. The significance level was set to α = 0.05. The coefficients of effect size were evaluated as follows: 0.2 ≤ d < 0.5—small effect size, 0.5 ≤ d < 0.8—medium effect size, and d ≥ 0.8—large effect size.

### Ethics approval

This study was performed in line with the principles of the Declaration of Helsinki. Approval was granted by the Ethics Committee of Palacký University Olomouc (No. 20/2021).

### Consent to participate

Informed consent was obtained from all individual participants included in the study.

## Results

The descriptive characteristics of the research participants are provided in Table 1. None of the research participants reported being married. The ASALL group comprised 45% of students and 55% of workers, while the CG group comprised 33% of students and 67% of workers (ASALL vs. CG, p = 0.45). There were no statistically significant differences (p ≥ 0.70 for all) in the anthropometric variables of age, height, weight, and BMI between the ASALL and CG groups. The participating ASALL became ill at a mean (± SD) age of 7.8 ± 5.0 years, and their treatment lasted for 16.3 ± 6.7 months. No ASALL underwent bone marrow transplantation. The mean time from diagnosis was 16.7 ± 5.4 years.

**Table 1:** Descriptive characteristics of the study sample (n = 41).

	Total *n* = 41		ASALL *n* = 20		CG *n* = 21		*p* value
	*Mdn*	*IQR*	*Mdn*	*IQR*	*Mdn*	*IQR*	
Age (years)	24.2	5.4	23.4	11.8	24.8	5.4	0.87
Female sex (% of *n*)	53.7		45		62		0.28^a^
**Anthropometric variables**							
Height (cm)	173.0	15.5	174	13.5	170.0	19	0.73
Weight (kg)	68.0	22.0	68.5	21	64.0	20	0.70
BMI	23.4	5.4	23.0	6.6	23.6	5.2	0.74
**Accelerometer use**							
Wear time (min/day)^b^	1439.4 ± 0.8		1439.4 ± 0.7		1439.5 ± 0.8		0.62^c^
Wake time (min/day)^b^	1019.8 ± 72.6		1021.9 ± 68.4		986.6 ± 60.6		0.79^c^
Wear time in wake time (%)	97.3		98.3		96.4		0.71^a^
Valid days^b^	6.8 ± 1.3		7.0 ± 1.3		6.5 ± 1.0		0.66^c^

*ASALL* Adult survivors of childhood acute lymphoblastic leukemia, *CG* Control group, *Mdn* Median, *IQR* Interquartile range, *BMI* Body mass index.

^a^Chi-square.

^b^Mean ± SD.

^c^t test.

The study participants wore the accelerometer for a mean of 1439.4 ± 0.8 min per day and had a mean of 6.8 ± 1.3 valid days. Statistical analysis revealed no significant differences between the ASALL and CG groups in terms of wear time, wake time, wear time during wake time, and number of valid days (all p > 0.05; data not shown).

The classification of the movement behavior data for the ASALL and CG are presented in Table 2. No significant differences in movement behavior were found between the ASALL and CG.

**Table 2:** Movement behavior in adult survivors of childhood acute lymphoblastic leukemia (ASALL) and the control group (CG).

	ASALL *n* = 20		CG *n* = 21		Difference	
	Mdn	IQR	Mdn	IQR	*p* value	d
**Movement behavior**						
SB (min/day)	711.1	124.5	636.1	135.9	0.26	0.35
LPA (min/day)	185.5	87.8	201.2	64.2	0.47	0.23
MPA (min/day)	132.1	78.9	147.3	83.3	0.25	0.36
VPA (min/day)	5.2	4.0	5.9	4.2	0.48	0.22

*ASALL* Adult survivors of childhood acute lymphoblastic leukemia, *CG* Control group, *Mdn* Median, *IQR* Interquartile range, *d* Effect size coefficient, *SB* Sedentary behaviour, *LPA* Light physical activity, *MPA* Moderate physical activity, *VPA* Vigorous physical activity.

A more detailed analysis reflecting the sex of the participants is displayed in Table 3. In both men and women, no significant differences in movement behavior were found between the ASALL and CG. There were also no significant differences (p ≥ 0.21 for all) in movement behavior between male and female participants within the ASALL or CG groups.

**Table 3:** Movement behavior in adult survivors of childhood acute lymphoblastic leukemia (ASALL) and the control group (CG) by sex.

	Male						Female					
	ASALL *n* = 11		CG *n* = 8		*p* value	d	ASALL *n* = 9		CG *n* = 13		*p* value	d
	Mdn	IQR	Mdn	IQR			Mdn	IQR	Mdn	IQR		
**Movement behavior**												
SB (min/day)	671.2	109.1	657.5	109.5	0.90	0.08	715.8	92.0	636.1	172.1	0.24	0.53
LPA (min/day)	201.4	71.5	199.1	79.6	0.60	0.27	181.9	75.5	201.2	83.5	0.29	0.47
MPA (min/day)	149.9	76.4	159.5	68.7	0.35	0.45	132.0	54.4	130.5	102.5	0.43	0.36
VPA (min/day)	5.9	2.0	6.0	8.6	0.72	0.19	4.2	8.9	5.9	4.2	0.70	0.19

*ASALL* Adult survivors of childhood acute lymphoblastic leukemia, *CG* Control group, *Mdn* Median, *IQR* Interquartile range, *d* Effect size coefficient, *SB* Sedentary behaviour, *LPA* Light physical activity, *MPA* Moderate physical activity, *VPA* Vigorous physical activity.

All the study participants met the recommendation of > 150 min of MPA per week (Table 4). The rate of compliance with the recommendation of > 75 min of VPA per week was substantially lower regardless of sex or participant group (ASALL and CG).

**Table 4:** Compliance with physical activity recommendations in adult survivors of childhood acute lymphoblastic leukemia (ASALL) and the control group (CG).

	ASALL		CG	
	*n*	*%*	*n*	*%*
**Physical activity recommendation**				
**> 150 min MPA/week**				
Male	11	100	8	100
Female	9	100	13	100
Total	20	100	21	100
**> 75 min VPA/week**				
Male	0	0	2	25
Female	2	22	1	8
Total	2	10	3	14

*ASALL* Adult survivors of childhood acute lymphoblastic leukemia, *CG* Control group, *SB* Sedentary behaviour, *LPA* Light physical activity, *MPA* Moderate physical activity, *VPA* Vigorous physical activity.

## Discussion

The main finding of this study was that the ASALL and the CG did not differ significantly in their movement behavior.

Although the ASALL group is less likely to achieve the health recommendations for PA and SB^7,12^, our study came to a surprising finding, as all the research participants (ASALL and CG) were able to achieve the recommended goal of > 150 min of MPA per week. The more stringent recommended goal for VPA (> 75 min per week) was rarely met.

When PA and SB were compared, there were no significant differences between the ASALL and CG, although a study by Stolley, Restrepo, & Sharp^23^ states that ASALL are likely to achieve lower levels of PA compared to the healthy population and not to meet the PA recommendations. One of the factors of the assumed reduction in PA may be the fact that although ASALL are aware of the possible effects of treatment and try to prevent them with a healthy lifestyle (healthy diet, non-consumption of alcohol or nonsmoking), some ASALL state that a fear of injury or concern for their health is the reason for their low level of PA or even the absence of PA^24^. In our study, we did not investigate attitudes toward PA, but they may play a role in movement behaviors and may be a subject of further investigation.

In our study, no significant differences in movement behavior were found in terms of the sex of the participants. Our findings on the differences in PA and SB between men and women are not consistent with the results of other studies that reported^25,26^ that women engaged in significantly less PA than men, and that emphasis should be placed on their support in participating in PA. For this reason, women aged 23–48 years who have survived acute lymphoblastic leukemia represent a patient population that is at risk of developing premature cardiovascular disease^26^. A study by Caru et al.^25^ reported differences in PA between young ASALL women and a healthy control group of women.

With regard to SB, we compared our findings with the study of Prince et al.^27﻿^, which reported that adult Canadians aged 18–34 years spent approximately 9–10 h per day in SB. From our study, the ASALL spent 711 min per day and the CG 636 min per day in SB, which is more than in the Canadian study. However, it is generally reported that healthy adults spend approximately 8.2 h per day in SB^28^. Conversely, the side effects of SB seem to be eliminated as soon as individuals are able to reach 60–75 min of MVPA per day^29^. This recommended goal was met by both the ASALL and CG in our study.

The results of our study indicate possible inconsistencies with those of other studies, which may suggest the need for a deeper understanding of the ASALL group and its PA and SB. Long-term and device-based monitoring of movement behavior can lead to an expansion of the spectrum of diagnostic approaches to patients, which can provide the key information needed to optimize the lifestyle and increase the quality of life of the target group.

The strengths of this study lie in device-measured data on PA and SB in the specific group of ASALL with long-term distance from treatment. Our study has several limitations that should be considered when interpreting the results. First, we found that the mean values of MPA in both the ASALL (132.1 min/day) and CG (147.3 min/day) groups were much higher than the recommended MPA of 150 min per week^16^. However, these recommendations were based on a questionnaire survey and primarily targeted activities in bouts of at least 10 min. Ramakrishnan et al.^30^ presented a study involving more than 90,000 adults in the United Kingdom. Participants were monitored using the same device, and data were evaluated using the same threshold as in our study. The results also showed high mean values for MPA, with a mean of 743.2 min per week (106.2 min per day). The similarity in results between our study and Ramakrishnan et al.'s study may suggest that the amount of MVPA derived from accelerometers and based on shorter epochs may not be fully compatible with current PA recommendations. Second, our study is based on a smaller non-representative research sample. Third, there was increased motivation for PA and awareness of freedom of movement in the participants after the long-lasting lockdown in the Czech Republic during the second wave of the COVID-19 pandemic. Finally, participation in the study was based on the self-interest of the participants. Thus, it is possible that those who were naturally more active also showed more interest in joining the study.

## Conclusions

The results of our study suggest that ASALL, even after suffering from that disease in childhood, reach comparable levels of PA and SB to their healthy peers. Both study groups consistently followed the health recommendations for PA. The device-based monitoring of PA and SB should be an integral part of monitoring the late effects of treatment.

## Data Availability

The datasets generated and/or analyzed during the current study are available from the corresponding author on reasonable request.
